# The hemodynamic interplay between pulmonary ischemia-reperfusion injury and right ventricular function in lung transplantation: a translational porcine model

**DOI:** 10.1152/ajplung.00281.2022

**Published:** 2023-09-19

**Authors:** Michaela Orlitová, Tom Verbelen, Anna E. Frick, Arno Vanstapel, Dieter Van Beersel, Sofie Ordies, Jan Van Slambrouck, Janne Kaes, Xin Jin, Walter Coudyzer, Stijn E. Verleden, Geert M. Verleden, Bart M. Vanaudenaerde, Dirk E. Van Raemdonck, Robin Vos, Laurens J. Ceulemans, Piet Claus, Arne P. Neyrinck

**Affiliations:** ^1^Department of Cardiovascular Sciences, KU Leuven, Leuven, Belgium; ^2^Department of Thoracic Surgery, University Hospitals Leuven, Leuven, Belgium; ^3^Department of Cardiac Surgery, University Hospitals Leuven, Leuven, Belgium; ^4^Department of Thoracic Surgery, Medical University of Vienna, Vienna, Austria; ^5^Department of Pathology, University Hospitals Leuven, Leuven, Belgium; ^6^Laboratory of Respiratory Diseases and Thoracic Surgery (BREATHE), Department of Chronic Diseases and Metabolism, KU Leuven, Leuven, Belgium; ^7^Department of Anesthesiology, University Hospitals Leuven, Leuven, Belgium; ^8^Department of Radiology, University Hospitals Leuven, Leuven, Belgium; ^9^Antwerp Surgical Training, Anatomy and Research Center, University of Antwerp, Antwerp, Belgium; ^10^Department of Respiratory Diseases, University Hospitals Leuven, Leuven, Belgium

**Keywords:** lung transplantation, porcine model, pulmonary ischemia-reperfusion injury, right ventricular function

## Abstract

Lung transplantation (LTx) is a challenging procedure. Following the process of ischemia-reperfusion injury, the transplanted pulmonary graft might become severely damaged, resulting in primary graft dysfunction. In addition, during the intraoperative window, the right ventricle (RV) is at risk of acute failure. The interaction of right ventricular function with lung injury is, however, poorly understood. We aimed to address this interaction in a translational porcine model of pulmonary ischemia-reperfusion injury. Advanced pulmonary and hemodynamic assessment was used, including right ventricular pressure-volume loop analysis. The acute model was based on clamping and unclamping of the left lung hilus, respecting the different hemodynamic phases of a clinical lung transplantation. We found that forcing entire right ventricular cardiac output through a lung suffering from ischemia-reperfusion injury increased afterload (pulmonary vascular resistance from baseline to end experiment *P* < 0.0001) and induced right ventricular failure (RVF) in 5/9 animals. Notably, we identified different compensation patterns in failing versus nonfailing ventricles (arterial elastance *P* = 0.0008; stroke volume *P* < 0.0001). Furthermore, increased vascular pressure and flow produced by the right ventricle resulted in higher pulmonary injury, as measured by ex vivo CT density (correlation: pressure *r* = 0.8; flow *r* = 0.85). Finally, RV ischemia as measured by troponin-T was negatively correlated with pulmonary injury (*r* = −0.76); however, troponin-T values did not determine RVF in all animals. In conclusion, we demonstrate a delicate balance between development of pulmonary ischemia-reperfusion injury and right ventricular function during lung transplantation. Furthermore, we provide a physiological basis for potential benefit of extracorporeal life support technology.

**NEW & NOTEWORTHY** In contrast to the abundant literature of mechanical pulmonary artery clamping to increase right ventricular afterload, we developed a model adding a biological factor of pulmonary ischemia-reperfusion injury. We did not only focus on the right ventricular behavior, but also on the interaction with the injured lung. We are the first to describe this interaction while addressing the hemodynamic intraoperative phases of clinical lung transplantation.

## Graphical abstract

**Figure:**
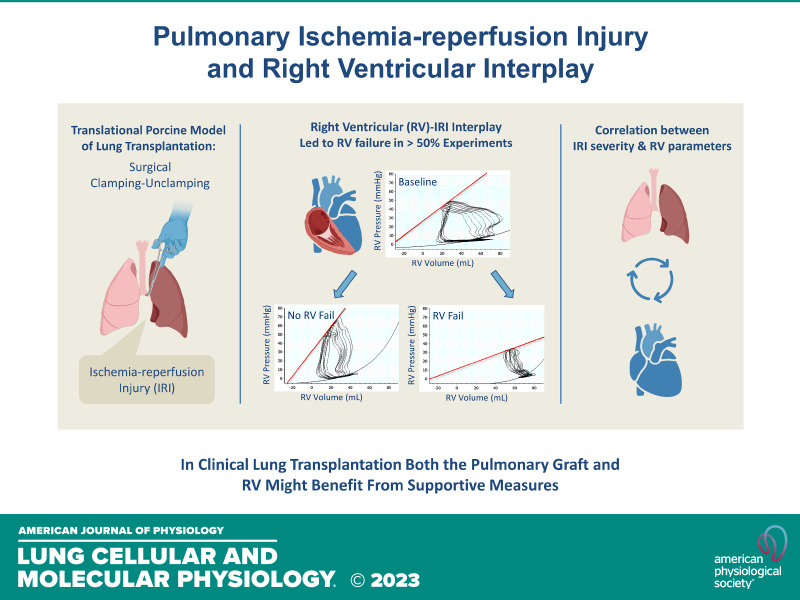

## INTRODUCTION

Lung transplantation (LTx) is a challenging procedure both on a technical and a physiological level. At the same time, it remains the only treatment option for end-stage lung disease. Within the first 72 h after reperfusion of the newly transplanted lungs, a clinical syndrome referred to as primary graft dysfunction (PGD) might occur. This complex syndrome of acute lung injury has been associated with increased morbidity and mortality immediately after LTx (1). PGD is a consequence of mechanical, immune, inflammatory, possible microbial, and other alterations associated with the implantation of a new organ, in which ischemia-reperfusion injury (IRI) plays a central role (2–4). IRI results from vascular endothelial dysfunction, alveolar epithelial injury, and inflammation (5, 6). However, other factors such as mechanical stress might contribute to this injury. The cessation and restoration of blood flow during the transplant process result in changes of shear stress at the endothelial lining. Eventually, these changes will lead to accumulation of pulmonary edema, which will increase pulmonary vascular resistance (PVR), impair oxygenation, and decrease lung compliance (6, 7).

The pathophysiological changes related to IRI might not only have repercussions on pulmonary graft function itself but also on right ventricular (RV) function and general hemodynamics of the patient. From a physiological point of view, the consequences of pulmonary dysfunction for the RV and vice versa are poorly described in the setting of IRI. First, increased PVR might dramatically increase RV afterload. Second, the performance and energy delivered by the RV might challenge the pulmonary vasculature at the mechanical level (8–10). Finally, many other factors including inflammatory and biological signaling originating from the process of IRI might have a yet unknown effect on the RV.

Apart from their mechanistic and scientific importance, answering these questions is highly relevant for daily clinical practice. The majority of LTx procedures are performed as a sequential single-LTx (SSLTx). This means that both diseased lungs are replaced by two new donor lungs in a sequential procedure (at the level of the individual lung). The sequence comprises four phases. During *phase 1*, one diseased lung is excluded from the circulation to be explanted. During this phase, the whole RV cardiac output (CO) is pumped through the remaining contralateral native diseased lung. This phase ends when the first lung is implanted and the graft is reperfused. During *phase 2*, both lungs are perfused (one graft and one diseased lung). In the new graft, the process of IRI starts at the moment of reperfusion. During *phase 3*, the surgeon will explant and replace the second diseased lung. Consequently, the whole RV CO is forced through the first newly transplanted graft only. During *phase 4*, both implanted lung grafts are perfused and the procedure is finalized.

It is obvious that this sequence is very challenging and that the recipient will depend on limited pulmonary and RV reserves at several timepoints during the transplantation procedure. Therefore, clinicians often use extracorporeal life support (ECLS) to aid the recipients circulation and gas exchange (11). To assess hemodynamic response and/or ECLS need, test clamping of a lung is performed before any irreversible surgical steps are taken.

The behavior of the RV in health and disease is very complex. Unlike the high resistance/low compliance systemic circulation, the pulmonary circulation is a low resistance/high compliance system. As a result, the left ventricle (LV) has a thick muscular wall with a contractility that is heavily dependent on preload via the Frank–Starling mechanism (Anrep effect). In contrast, the RV has a thin muscular wall, its contractility is less dependent on preload and has less capacity to deal with sudden increases in afterload (9, 12). If RV pressure rises, such as during acute afterload increase caused by surgical clamping, RV stroke volume (SV) decreases much faster compared with the systemic circulation (8). Furthermore, the pressure overload leads to increased RV contractility based on homeometric autoregulation that seems to be more driven by sympathetic stimulation than LV Anrep effect (13). However, if the afterload increases even further, the RV compensation mechanism might become exhausted. Decrease in RV function and RV dilatation leads eventually to impairment of LV function as well (10, 14, 15).

The overall aim of this paper is to further unravel RV function in a specific setting of pulmonary IRI. This will be performed in a porcine model of in situ pulmonary clamping and unclamping, mimicking the first three phases of SSLTx.

Our hypothesis is dual. First, we assumed that IRI of the lung might have an impact on RV function, especially when combined with mechanical clamping of the contralateral lung. Second, we hypothesized that hemodynamic changes occurring during different phases of LTx will have repercussions on the transplanted graft itself.

## MATERIALS AND METHODS

This experimental porcine study (Strain: TN70, Topigs Norsvin) was approved by the Ethics Committee on Animal Research KU Leuven (P0115/2020). All animals received human care in accordance with Principles of Laboratory Animal Care, formulated by the National Society for Medical Research and Guide for the Care and Use of Laboratory Animals, prepared by the Institute of Laboratory Animal Resources and published by the National Institutes of Health, USA (NIH Publication No. 86-23, revised 1996).

### Experimental Protocol

#### Anesthesia maintenance.

Eighteen male pigs (*n* = 18) were randomly divided into two groups: *Group 1* was the clamping (CLA; *n* = 9) group and *group 2* was the control (CON; *n* = 9) group. Sedation of the animals was performed with an intramuscular injection of 5 mg/kg Zoletil 100 (Virbac, Carros, France) and 3 mg/kg Xyl-M 2% (VMD, Arendonk, Belgium), anesthesia was maintained with 10 mg/kg/h propofol, 20 μg/kg/h fentanyl, and intermittent boli of pancuronium 4 mg for muscle relaxation and 20 mg lidocaine for rhythm control. Lactated Ringer’s was continuously administered (8 mL/kg/h) to compensate fluid losses and was given in bolus whenever appropriate to compensate for blood loss, with respect to the hemoglobin levels. All animals received low-dose vasopressor support to maintain mean arterial blood pressure (ABP) > 50 mmHg (norepinephrine 0.01–0.05 µg/kg/min). Animals were intubated in a supine position with a 8.0-mm endotracheal tube and ventilated with a tidal volume (TV) of 8 mL/kg, positive end-expiratory pressure (PEEP) of 5 cmH_2_O and a fraction of inspired oxygen ($FI_{O_{2}}$) of 30%. Respiratory rate (RR) was adjusted to end-tidal carbon dioxide levels of 40–45 mmHg.

#### Surgical procedures.

A lateral right neck incision was performed for invasive monitoring of ABP in the carotid artery and central venous pressure monitoring (CVP) in the internal jugular vein. Under X-ray visualization (GE OEC 7900 fluorostar compact), a 8.5-Fr vascular sheath was inserted through the external jugular vein with the tip in the right atrium to facilitate the RV conductance catheter. Next, a 11-Fr vascular sheath was placed in the left femoral vein using Seldinger’s technique with ultrasound guidance to facilitate introduction of a 9-Fr balloon catheter for preload reduction at the level of the inferior caval vein (ICV). A mini-laparotomy was performed to insert a bladder catheter and the body temperature was measured with a rectal probe.

**Figure 1.**
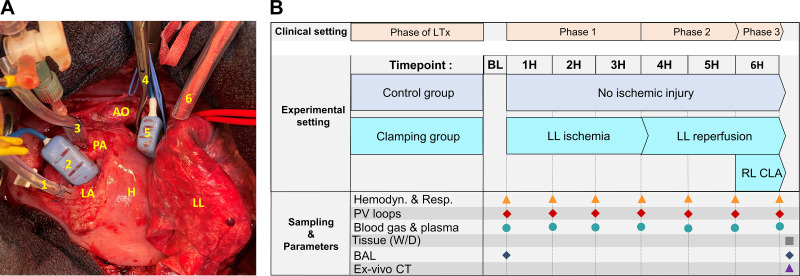
*A*: open chest experimental setup demonstrating the instrumentation. *B*: clinical lung transplantation (LTx) setting vs. experimental protocol: overview of sampling and parameter recording. AO, aorta; H, heart; LA, left atrium; LL – left lung; PA, pulmonary artery.1—LA pressure catheter; 2—PA flow probe; 3—PA pressure catheter; 4—vascular clamp on left PA and left main bronchus; 5—left PA flow probe; 6—vessel loop around Right PA enabling its closure during 6H; BL, baseline; 1H, 1 hour; 2H, 2 hours; 3H, 3 hours; 4H, 4 hours; 5H, 5 hours; 6H, 6 hours; RL CLA, right PA clamping; Hemodyn., hemodynamic parameters, Resp., respiratory parameters; PV, pressure-volume; *W*/*D*, wet-to-dry ratio; BAL, bronchoalveolar lavage; CT, computed tomography.

The pig was turned to a right lateral decubitus position and a left thoracotomy in the 4th intercostal space was performed with the removal of *ribs 3*–*5*. After transection of the pulmonary ligament and the left hemi-azygos vein, dissection of the left main bronchus and left and right pulmonary artery (PA) was performed, to facilitate the control of the structures. Next, the pericardium was opened and flow probes (Transonic Systems Inc., Ithaca, NY) were placed around the main PA trunk and the left PA (LPA) to determine the flow toward the left and right lung separately during the protocol. All animals received 25,000 IU of unfractionated heparin (LEO Pharma, Ballerup, Denmark). Following, a pressure monitoring line was introduced in the main PA and the left atrium (LA). The open chest experimental setup can be seen in Fig. 1*A*. Next, a conductance catheter (Ventri-cath 507, Millar Instruments, Houston, TX) to measure the pressure-volume (PV) loops was placed in the RV with X-ray guidance. After performing all baseline measurements and sampling as described later, the animals were randomized into CON or CLA group.

Animals in the CON group were monitored and sampled during the period of 6 h. No ischemic injury was induced. In the CLA group, the LPA and the left main bronchus were clamped for 3 h (and the ventilation was adjusted to TV of 6 mL/kg) to both induce warm ischemic injury and simulate *phase 1* of the LTx procedure as described earlier. Afterward, the injured left lung (LL) was reperfused for 2 h together with the right native lung (reflects *phase 2* of LTx procedure). To simulate *phase 3*, the contralateral noninjured right lung (RL) was clamped for an additional hour. At that moment, the RV CO was forced through the post-ischemic LL only. At the end of the experiment, the animals were euthanized in both groups under anesthesia by exsanguination.

RV failure was defined as death of the animal before completing the protocol or as an acute decrease of RV CO > 50%.

#### Recording and analyzing RV pressure-volume loops.

The principle of PV loop recording is the real-time beat-to-beat measurement of ventricular pressures and volumes using a conductance catheter. In addition, reducing the preload (by inflating the ICV balloon) allows registration of contractility parameters.

Prior to conductance catheter placement, pressure calibration was performed as well as the correction for the animals’ blood resistivity and conductivity with Rho calibration cuvette (ADInstruments, Oxford, UK). After placement in the RV, myocardial parallel conductance was excluded with hypertonic saline (2 × 15 mL 7% NaCl) injection. Every hour, three preload reduction maneuvers in apnea were performed consecutively with a period of recuperation in between. During the last hour of the experiment, PV loops were recorded continuously without cessation of breathing. Data were recorded through PowerLab 8/35 (ADInstruments, Oxford, UK), connected to a transducer amplifier (Millar Instruments, Houston, TX) and analyzed using LabChart software (ADInstruments, Oxford, UK).

The following parameters were analyzed: end-systolic and end-diastolic pressure, stroke volume (SV), stroke work (SW), end-systolic elastance (Ees), arterial elastance (Ea). SW refers to the mechanical work done by the ventricle to eject SV. Ees was considered in combination with the position of the volume axis intercept (*V*_0_), as a measure of RV contractility (16). Ea represents the mechanical characteristics of the pulmonary vasculature and is a major determinant of afterload opposing RV ejection. Energy transfer between RV and pulmonary circulation is characterized by RV-PA coupling (Ees/Ea).

### Data from Last Experimental Hour

Single-beat measurements were used for analysis during the last experimental hour to capture their evolution in time or if the RV failed before performing the final preload reduction.

A detailed analysis of the CLA group was performed. A discrimination was made between the animals that did develop RV failure (RVF) and those who did not (NO RVF). The timepoints for analysis were chosen according to their importance toward the clinical LTx. All parameters were measured before and after right pulmonary artery (RPA) clamping to differentiate between the changes caused purely by mechanical clamping and the ones caused by the presence and/or increase of lung injury. Time intervals of 5 and 10 min are relevant in relation to the test clamping before lung extraction (as described earlier). Timepoint of peak SW reflects on the maximal ejection work produced by RV. End-experiment value was measured 60 min after RPA clamping (NO RVF) or at the point of hemodynamic collapse (RVF).

### Sampling

Hemodynamic and ventilatory (lung compliance) parameters were continuously recorded using ICM+ (Cambridge Enterprise, Cambridge, UK). Hourly, blood was taken and centrifuged to obtain plasma samples for troponin-T and cytokine analysis. Furthermore, the blood gas analysis was performed by obtaining samples from the catheter in the carotid artery, the pressure line in the PA, and from direct puncture of the left and right lower lobe pulmonary veins. This allowed discriminating the oxygenation capacity of both lungs. At the end of the experiment, the whole lung block was extracted. Samples from both lower lobes were taken for wet-to-dry (*W*/*D*) ratio assessment to quantify the amount of pulmonary edema. The weight of the samples was measured and compared before and after a period of 72 h in an 80°C oven.

Ex vivo computed tomography (CT) scan (Siemens Healthcare, Erlangen, Germany) of inflated lungs was performed and HOROS program [DICOM Viewer, v.3 (GPL-3.0)] was used to measure parenchymal density as a marker of pulmonary injury (17).

After CT scanning, tissue sampling for histology assessment (hematoxylin-eosin) was performed. All samples were taken from the lower lobe at the center of the parenchyma. The samples were scored in the presence of interstitial edema, intra-alveolar edema, hemorrhage, interstitial cell infiltration, intra-alveolar neutrophile infiltration, and in the presence of hyaline membranes. Each feature was assessed using a semiquantitative scoring system of 0–3 (0—absent; 1—mild; 2—moderate; 3—severe) and the total injury score was reported (18). Scoring was performed by a pathologist blinded for the origin of the samples. Troponin-T was measured at baseline (BL) and at last two experimental hours (before RL clamping: 5H and end experiment: 6H). For cytokine analysis, plasma level of interleukin (IL-) 1β, IL-12p40, IL-4, IL-6, IL-8, IL-10, interferon (IFN) α, IFN-γ, and tumor necrosis factor-α (TNF-α) were measured at baseline and at last experimental hour (6H). A porcine multiplex enzyme-linked immunosorbent assay was used according to the manufacturer’s protocol (Thermo Fischer Scientific, Vienna, Austria).

Summary of performed sampling and correlation of the experimental protocol with the hemodynamic events of clinical LTx are visualized in Fig. 1*B*.

### Statistical Analysis

All data were analyzed in GraphPad Prism 9 (GraphPad Software Inc, La Jolla, CA). Physiological parameters were considered as normally distributed, and therefore the data are presented as means ±SD. Groups were compared by unpaired *t* test. Where applicable, values were compared only between time points (repeated measures over time) or also between both study groups using two-way ANOVA for repeated measures and post hoc multiple comparison test Sidak (x). To investigate correlation between CT measured density and hemodynamic parameters, Pearson *r* correlation coefficient was calculated. Differences were considered significant if *P* value ≤0.05.

## RESULTS

We included 18 animals, but two experiments from the CON group failed due to sickness of the animal (*n* = 1) and a surgical complication (*n* = 1). Therefore, the analysis was performed with *n* = 9 in CLA and *n* = 7 in CON. The average weight in CON group was 49.4 (±8.3) kg and 50.8 (±4.3) kg in CLA group and was not significantly different (*P* = 0.65).

RVF occurred in five animals of the CLA group and developed during the final experimental hour when the RL was clamped (*phase 3*). Of these five animals, two animals developed acute decrease of RV CO > 50% and three animals did arrest prematurely before completion of the protocol. No animal in CON group developed RV failure.

### Assessment of Lung Injury

**Figure 2.**
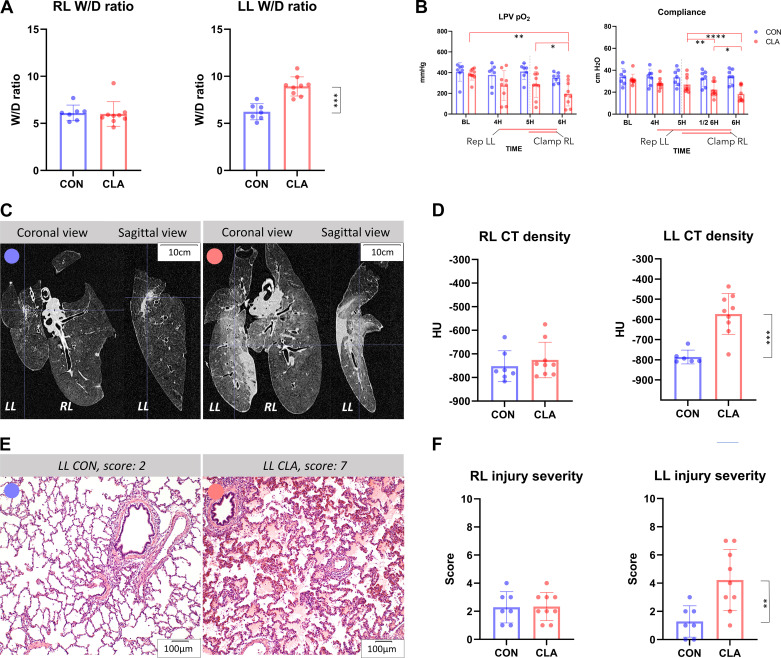
Lung injury assessment. *A*: wet-to-dry (W/D) ratio reflecting left lung (LL) injury. *B*: physiological parameters: left pulmonary vein (LPV) Po_2_ measured at 100% fraction of inspired O_2_ at baseline (BL) and during reperfusion; lung compliance at baseline and during reperfusion, both reflecting LL injury. *C*: computed tomography (CT) scan, example of increased density in clamping experimental group (CLA) compared with control experimental group (CON). *D*: severity of LL injury quantified by CT-measured parenchymal density. *E*: example of LL histology slide scored as 2 (CON) and 7 (CLA). *F*: histological lung injury severity score confirming severe LL injury. *P < 0.05; **P < 0.01; ***P < 0.001; ****P < 0.0001. Data presented as individual values and means ±SD; *t* test (*A*, *D*, and *F*) and two-way ANOVA (*B*) was used; CON (*n* = 7), CLA (*n* = 9). HU, Hounsfield units; RL, right lung; LPV, left pulmonary vein; Po_2_, partial pressure of oxygen; 4H, 4 hours; 5H, 5 hours; ½ 6H, halfway through 6H; 6H, 6 hours; CON (blue circle), control group; CLA (orange circle), clamping group; gray line (in *B*) indicates change of hemodynamic phase.

Figure 2 demonstrates lung injury quantification using a *W*/*D* ratio calculation, physiological, radiological, and morphological assessment. *W*/*D* ratio (Fig. 2*A*) showed no significant difference in the presence of lung edema in the RL between CON and CLA groups (*P* = 0.34), whereas a significant difference was found in the LL between the groups (*P* = 0.0003). Physiological parameters (Fig. 2*B*) showed deterioration of LL oxygenation function and overall lung compliance during reperfusion. Partial pressure of oxygen (Po_2_) in left pulmonary vein (LPV) remained stable as opposed to baseline (BL) at 4H and 5H (*P* = 0.32, *P* = 0.29, respectively) and decreased significantly only at 6H (*P* = 0.0043). The progressive deterioration of LL function is also reflected by continuous decrease in lung compliance between 5H and both half time of 6H (1/2 6H) and 6H (*P* = 0.0034, *P* < 0.0001, respectively). CT-measured parenchymal density (Fig. 2, *C* and *D*) showed comparable results for RL (*P* = 0.41) and significant difference for LL when comparing CON and CLA groups (*P* = 0.0008). Notably, the range of injuries was wider than reflected by the *W*/*D* ratio. Morphological assessment (Fig. 2, *E* and *F*) showed the same pattern of results with no significant difference for RL (*P* = 0.94) but significant difference for LL (*P* = 0.0087).

### Plasma Cytokine Analysis

Inflammatory response to induced injury was quantified by analyzing cytokines. From all measured cytokines (Supplemental Fig. S1, *A–I*), only IL-6 showed a significant difference at 6H between CON and CLA groups (*P* = 0.0004). A difference between BL and 6H in CON group was measured in IL-6, IL-1β, and TNF-α, respectively (*P* = 0.0004, *P* = 0.038, *P* = 0.0268). In CLA group, a difference between BL and 6H was measured in IL-6, IL-1β, IL-10, and TNF-α, respectively (*P* = <0.0001, *P* = 0.0012, *P* = 0.0007, *P* = 0.0033).

### Hemodynamics

Table 1 lists the measured parameters. There were no significant differences in any baseline (BL) parameters between the CON and CLA groups. The tables for all other timepoints: *hour 1*–*6* (1H, 2H, 3H, 4H, 5H, 6H) can be found in Supplemental Material (Supplemental Tables S1–S6).

**Table 1.** Baseline parameter comparisons between experimental groups show no difference

Variable	CONTROL Group (*n* = 7)	CLAMPING Group (*n* = 9)	*P* Value	Significance
Weight, kg	49.4 ± 8.3	50.8 ± 4.4	0.65	ns
HR, beats/min	82 ± 16	85 ± 14	0.32	ns
mABP, mmHg	113 ± 22	112 ± 20	0.93	ns
mPAP, mmHg	19 ± 2.8	21 ± 2.6	0.12	ns
CVP, mmHg	2 ± 2.5	3 ± 3.1	0.56	ns
LAP, mmHg	3 ± 1.5	4 ± 3.5	0.50	ns
Temp, °C	38.2 ± 0.8	38.2 ± 0.5	0.98	ns
Pulse oximetry, %	96 ± 1.3	97 ± 1.6	0.98	ns
PVR, dyn/s/cm^−5^	310 ± 65	310 ± 75	0.99	ns
Flow probes				
RV CO, L/min	4.1 ± 0.75	4.1 ± 0.61	0.89	ns
Flow LPA, L/min	1.2 ± 0.33	1.2 ± 0.6	0.82	ns
Blood gas-analysis				
pH, mmHg	7.37 ± 0.04	7.37 ± 0.03	0.83	ns
${\text{Pa}}_{{\text{O}}_{\text{2}}}$, mmHg	444 ± 37.2	437 ± 45.5	0.74	ns
${\text{Pa}}_{{\text{CO}}_{\text{2}}}$, mmHg	44 ± 5.2	44 ± 4.1	0.98	ns
LPV Po_2_, mmHg	395 ± 89.8	395 ± 32.5	0.99	ns
RPV Po_2_, mmHg	367 ± 78	376 ± 71	0.79	ns
PV-analysis				
RV ESP, mmHg	33.7 ± 2.3	38.1 ± 5.4	0.06	ns
RV EDP, mmHg	4.5 ± 1.89	6.7 ± 2.5	0.08	ns
RV SV, mL	46.4 ± 9.03	44.9 ± 9.42	0.74	ns
RV Ea, mmHg/mL	0.76 ± 0.2	0.9 ± 0.21	0.18	ns
RV Ees, mmHg/mL	0.44 ± 0.19	0.47 ± 0.22	0.75	ns
RV Ees/Ea	0.64 ± 0.36	0.54 ± 27	0.54	ns
RV SW, mmHg·mL	1,456 ± 503.1	1,497 ± 332.2	0.84	ns

Values are means ± SD (*t* test). CVP, central venous pressure; Ea, arterial elastance; Ees, end-systolic elastance; EDP, end-diastolic pressure; ESP, end-systolic pressure; HR, heart rate; LAP, left atrial pressure; LPA, left pulmonary artery; LPV Po_2_, left pulmonary venous partial pressure of oxygen; mABP, mean arterial blood pressure; mPAP, mean pulmonary artery pressure; ${\text{Pa}}_{{\text{CO}}_{\text{2}}}$, arterial partial pressure of carbon dioxide; ${\text{Pa}}_{{\text{O}}_{\text{2}}}$, arterial partial pressure of oxygen; pH, potential of hydrogen; PVR, pulmonary vascular resistance; RPV Po_2_, right pulmonary venous partial pressure of oxygen; RV CO, right ventricular cardiac output; SV, stroke volume; SW, stroke work; Temp, temperature.

### LV Function and Systemic Circulation

Mean ABP (Supplemental Fig. S2*A*) decreased throughout the experiment in both control and clamping groups. In the clamping group, the decrease was statistically significant at 4H (*P* = 0.0104), 5H (*P* = 0.0204), and 6H (*P* = 0.0247) when compared with BL. Systemic vascular resistance (Supplemental Fig. S2*B*) was comparable in both groups with the exception of 6H when it significantly decreased in CLA group. Both left atrial pressure and central venous pressure (Supplemental Fig. S2, *C* and *D*) were comparable between both groups at all time timepoints.

### Pulmonary Hemodynamics, Preload and Afterload

**Figure 3.**
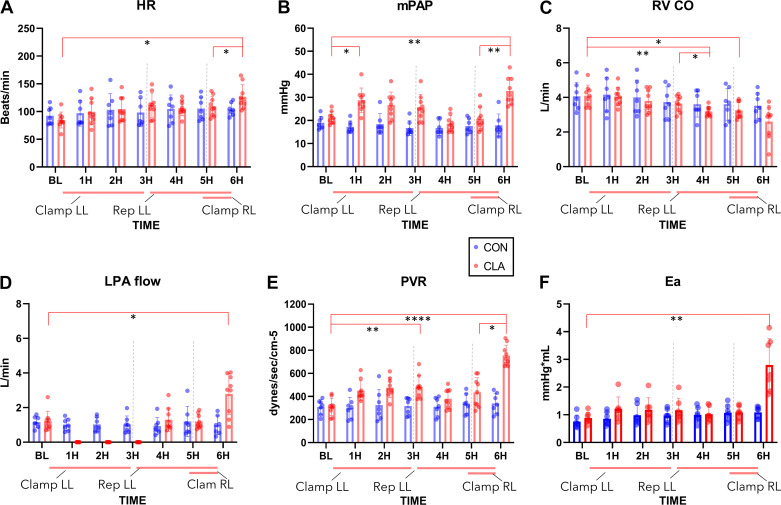
Pulmonary hemodynamics and afterload. *A*: HR, heart rate increased at 6H. *B*: mPAP, mean pulmonary artery pressure increased both as a response to left lung (LL) (1H) and right lung (RL) clamping (6H). *C*: RV CO, right ventricular cardiac output decreased throughout the experiment. *D*: LPA flow, left pulmonary artery flow increased after RL clamping. *E*: PVR, pulmonary vascular resistance increased in a same trend as mPAP. *F*: Ea, arterial elastance increased at 6H as a response to mechanical clamping and pulmonary injury; BL, baseline; 1H, 1 Hour; 2H, 2 Hours; 3H, 3 Hours; 4H, 4 Hours; 5H, 5 Hours; 6H, 6 Hours; Clamp LL, left lung clamped after BL measurement; Rep LL, left lung reperfusion; Clamp RL, right lung clamped; Gray lines indicates change of hemodynamic phase. *P < 0.05; **P < 0.01; ****P < 0.0001. Data presented as individual values and means ±SD, values for 1H–6H are an average of the whole experimental hour; two-way ANOVA was used. CON (*n* = 7), CLA (*n* = 9).

The heart rate (HR; Fig. 3*A*) in CLA group increased from BL to 6H and from 5H to 6H (*P* = 0.019, *P* = 0.041, respectively). The mean pulmonary artery pressure (mPAP; Fig. 3*B*) in CLA group increased significantly between BL and 1H (*P* = 0.027), normalized during reperfusion and increased again as compared with BL at 6H (*P* = 0.0053). Clamping of the RPA was followed by significant increase between 5H and 6H (*P* = 0.0011). A decrease in RV CO (Fig. 3*C*) was noted in CLA group, from BL to 4H and 5H (*P* = 0.0033, *P* = 0.02, respectively). Also, the reperfusion of the LL was followed by CO decrease between 3H and 4H (*P* = 0.036). As intended by the experimental protocol, there was no left pulmonary artery flow (Fig. 3*D*) during LL clamping, whereas at 6H it increased as opposed to BL (*P* = 0.0346).

The PVR values (Fig. 3*E*) were significantly increased in the CLA group at multiple timepoints. During LL clamping, the increase from BL was less pronounced (*P* = 0.0032) compared with 6H (*P* = < 0.0001). Consequently, an increase from 5H to 6H was present (*P* = 0.017). Interestingly, RV Ea (Fig. 3*F*) did not increase significantly during the period of LL clamping and returned to baseline values at 4H and 5H, only at 6 h it increased significantly compared with BL (*P* = 0.0063).

### RV Function

**Figure 4.**
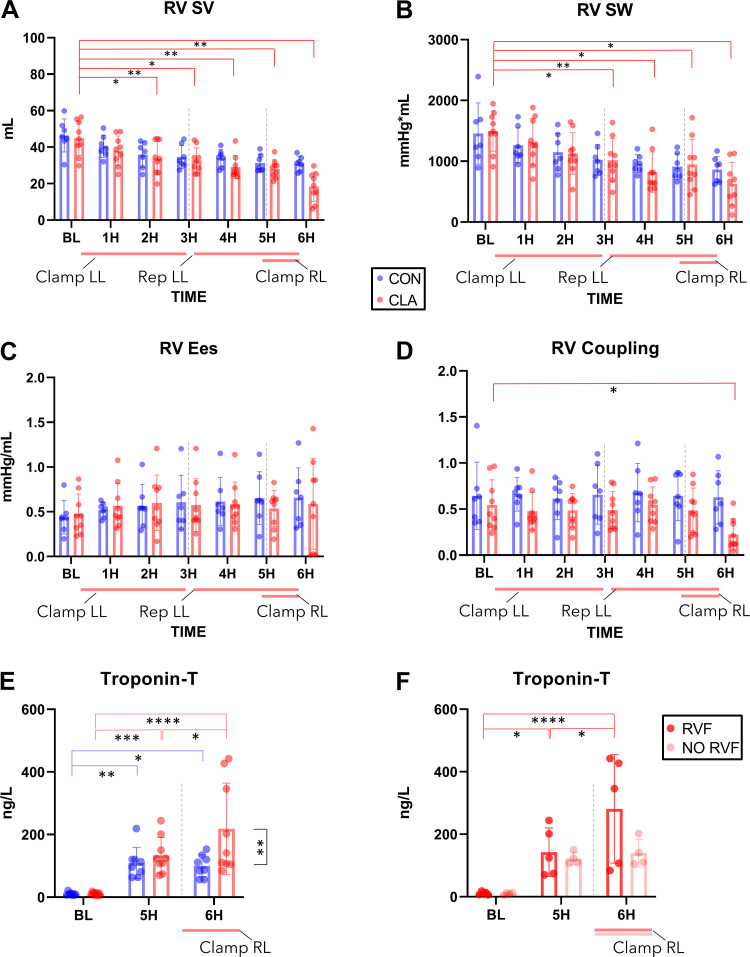
Conductance catheter derived analysis of right ventricular (RV) function and troponin-T plasma levels. *A*: RV SV, right ventricular stroke volume decreased through the experiment. *B*: RV SW, right ventricular stroke work decreased through the experiment. *C*: RV Ees, right ventricular end-systolic elastance showed no difference in time or between groups. *D*: RV Coupling, right ventricular-pulmonary artery coupling decrease at 6H suggesting uncoupling. *E*: Troponin-T increase in both control (CON) and clamping (CLA) groups with significant difference between groups at 6H; *(F)* Troponin-T increased in time in experiments resulting in right ventricular failure (RVF) but did not reach significance in experiments not resulting in right ventricular failure (NO RVF). BL, baseline; 1H, 1 Hour; 2H, 2 Hours; 3H, 3 Hours; 4H, 4 Hours; 5H, 5 Hours; 6H, 6 Hours; Clamp LL, left lung clamped after BL measurement; Rep LL, left lung reperfusion; Clamp RL, right lung clamped. Gray line indicates change of hemodynamic phase. *P < 0.05; **P < 0.01; ***P < 0.001; ****P < 0.0001. Data presented as individual values and means ±SD; two-way ANOVA was used. CON (*n* = 7), CLA (*n* = 9); RVF (*n* = 5), NO RVF (*n* = 4).

During the course of the experiment, RV SV (Fig. 4*A*) decreased in both CON and CLA groups. In the CLA group, a difference was found between BL and all timepoints starting from 2H (2H *P* = 0.0284, 3H *P* = 0.0033, 4H *P* = 0.0173, 5H *P* = 0.0051, 6H *P* = 0.0084). Similarly, a decrease in SW (Fig. 4*B*) was seen in both groups. A significant difference was observed in the CLA group between BL and all timepoints starting from 3H (3H *P* = 0.0115, 4H *P* = 0.0025, 5H *P* = 0.0445, 6H *P* = 0.0125). No significant difference was present in CON group for both SV and SW. Looking at RV contractility, Ees (Fig. 4*C*) did not significantly change during the course of the experiment. Also, *V*_0_ did not change between the groups (Supplemental Table S7). There was no significant difference in ventriculo-arterial coupling observed between the groups, apart from a decrease at 6H (*P* = 0.026), suggesting uncoupling during this period (Fig. 4*D*).

Troponin-T plasma analysis was performed to assess the amount of cardiac ischemic injury present (Fig. 4*E*). In both CON and CLA groups, a significant increase of troponin-T was observed between BL and 5H or 6H, respectively (CON 5H *P* = 0.0054, 6H *P* = 0.0124; CLA 5H *P* = 0.0003, 6H *P* = <0.0001). Moreover, in CLA group troponin-T levels increased after RL clamping (*P* = 0.0123). At the end of the experiment (6H), a significant difference was measured between CON and CLA groups (*P* = 0.0037). Next, in the CLA group, troponin-T values were compared between experiments resulting in RV failure (RVF) and those that did not (NO RVF; Fig. 4*F*). No statistically significant difference was found between RVF and NO RVF groups in BL, 5H, and 6H, respectively (*P* = >0.9999, *P* = 0.9792, *P* = 0.0631). In RVF group, a difference was observed between BL and 5H or 6H, respectively (*P* = 0.0253, *P* = <0.0001) and between 5H and 6H (*P* = 0.018). In NO RVF group, no statistical significance was reached either between BL and 5H or 6H (5H *P* = 0.1002, 6H *P* = 0.0509) or between 5H and 6H (*P* = 0.9791).

### Detailed Analysis of Last Experimental Hour (6H) in CLA Group: Failing (RVF) versus Nonfailing (NO RVF) Ventricles

**Figure 5.**
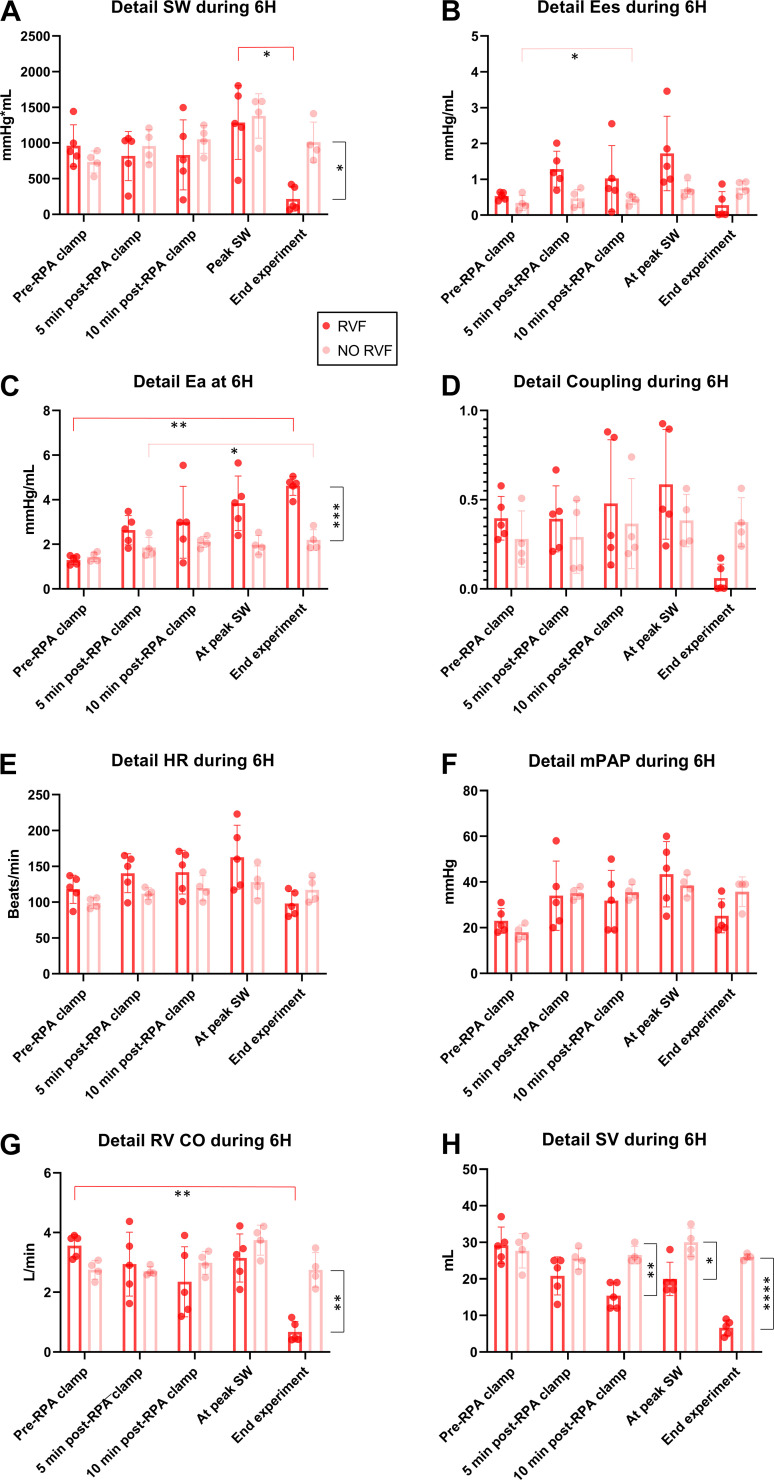
CLA group—comparing failed (RVF) vs. non-failed (NO-RVF) ventricles during the last experimental hour (6H). *A*: SW, stroke work decreased in RVF compared to No-RVF group after reaching peak. *B*: Ees, end-systolic elastance had an increasing tendency in RVF group to the point of failure resulting in Ees decrease. *C*: Ea, arterial elastance showing a different pattern between two groups. *D*: right ventricular-pulmonary artery coupling demonstrating a trend toward difference in two groups at end experiment. *E*: HR, heart rate remained comparable in both groups. *F*: mPAP, mean pulmonary arterial pressure remained comparable in both groups. *G*: CO, cardiac output decreased at end experiment in RVF group reflecting RV failure. *H*: SV, stroke volume demonstrating a different pattern between two groups from 10 min after right pulmonary artery (RPA) clamp. CLA, clamping. *P < 0.05; **P < 0.01; ***P < 0.001; ****P < 0.0001. Data presented as individual values and means ±SD; two-way ANOVA was used; RVF (*n* = 5), NO RVF (*n* = 4).

Since RVF occurred in some animals during the last experimental hour, we performed a detailed analysis of ventricular function between RVF (*n* = 5) versus NO-RVF (*n* = 4) groups. The evolution of SW throughout the last hour (Fig. 5*A*) showed no significant difference in mechanical work produced by the ventricles between RVF and NO RVF groups, till the moment of hemodynamic collapse in RVF group (*P* = 0.025). This was also reflected by significant SW drop between peak SW value and end experiment in RVF group (*P* = 0.37). The actual peak SW values and timing of their occurrence within 6H can be found in Supplemental Material (Supplemental Table S8).

When comparing the contractility (Ees) between the two groups, following was noted: although not statistically significant compared with NO RVF (*P* = 0.17), all ventricles in RVF group produced a contractility peak (reflecting SW peak) before the failure (Fig. 5*B*). As reflected by Ea (Fig. 5*C*), a difference in afterload increase was observed between the two groups. Whereas Ea was comparable with pre-RPA clamping (*P* = 0.89), a significant difference was found between the two groups at the end point (*P* = 0.0008). In the RVF group, the difference between pre-RPA clamping and end point was also significant (*P* = 0.0019). In the NO RVF group, a difference was measured between the point of 5 min post-RPA clamping and end experiment (*P* = 0.014). There was a trend toward a difference in ventriculo-arterial coupling (Fig. 5*D*) between the groups at the end of the experiment (*P* = 0.058), reflecting the outcome (RVF/NO RVF).

When assessing hemodynamic parameters standardly measured during LTx, no statistically significant difference between the two groups or between the timepoints was found in HR and mPAP (Fig. 5, *E* and *F*). RV CO (Fig. 5*G*) decreased in the RVF group compared with NO RVF only at the end of the experiment, when RVF developed (*P* = 0.0093). This was also reflected in RVF group when comparing pre-RPA clamp with end experiment (*P* = 0.0084). Interestingly, a difference in SV (Fig. 5*H*) was observed between the two groups. In the RVF group, a steady decrease was noted with significant differences at 10 min after RPA clamping (*P* = 0.0041), at peak SW (*P* = 0.048) and was most pronounced at end experiment point (*P* < 0.0001).

### Correlating Pulmonary Hemodynamics to the Severity of Lung Injury

**Figure 6.**
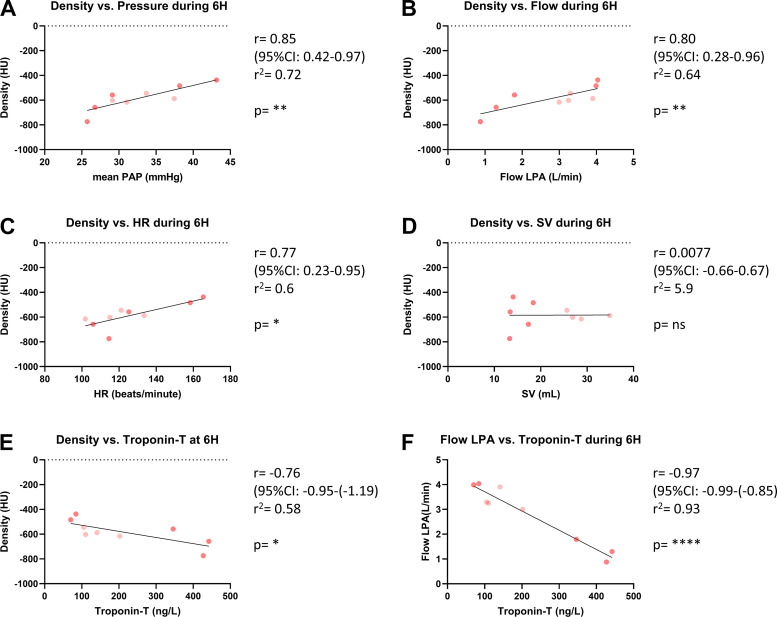
Correlating hemodynamic parameters, severity of pulmonary injury and cardiac ischemic injury in clamping (CLA) group [including experiments resulting in right ventricular failure (RVF) + experiments not resulting in RVF (NO RVF)]. *A*: correlation between mean pulmonary artery pressure (mPAP) and computed tomography (CT)-measured pulmonary density. *B*: correlation between left pulmonary artery (LPA) flow and CT-measured pulmonary density. *C*: correlation between heart rate (HR) and CT-measured pulmonary density. *D*: no correlation found between stroke volume (SV) and CT-measured pulmonary density. *E*: negative correlation between cardiac ischemic injury (Troponin-T) and CT-measured pulmonary density. *F*: strong negative correlation between left pulmonary artery (LPA) flow and cardiac ischemic injury (Troponin-T). Orange circles, experiment resulted in RVF; tan circles, experiment did not result in RVF. HU, Hounsfield Units; LPA Flow, flow in left pulmonary artery; RVF, right ventricular failure. *P < 0.05; **P < 0.01; ****P < 0.0001. Data presented as individual values; Pearson *r* was used for correlation assessment; RVF (*n* = 5), NO RVF (*n* = 4).

The impact of pulmonary hemodynamics on the amount of LL injury in CLA group during the last experimental hour was studied. A significant correlation was found between CT-measured density and average mPAP (Fig. 6*A*; *P* = 0.0038, *r* = 0.85), as well as the average flow in LPA (Fig. 6*B*; *P* = 0.0099, *r* = 0.8) during 6H.

To establish the underlying driving factor of this correlation, the HR and SV were also correlated to LL CT-measured density. The mean HR during the last hour was predictive of the severity of lung injury (Fig. 6*C*; *P* = 0.014, *r* = 0.77) whereas the mean SV was not (Fig. 6*D*; *P* = 0.98, *r* = 0.0077).

The interaction between amount of LL and RV injury was studied by correlating LL CT-measured density and troponin-T at the end of the experiment. A significant negative correlation was found (Fig. 6*E*; *P* = 0.0179, *r* = −0.76). Interestingly, troponin-T values were also strongly negatively correlated with the average flow in LPA during 6H (Fig. 6*F*; *P* = <0.0001, *r* = 0.97).

## DISCUSSION

In this study, we investigated the interplay between pulmonary IRI and RV function in the context of SSLTx. Our model allowed to dissect the factors leading to PGD and focus on the intraoperative hemodynamics of a SSLTx procedure, including the effects of surgical clamping and unclamping. RV afterload increase leading to RVF has been previously studied in other animal models (19). However, RV function in the specific setting of pulmonary IRI has not been thoroughly studied before.

Our main findings are that increased afterload (Ea) due to IRI and clamping is an important parameter that challenges the compensation ability of the RV. Ventricles were prone to fail when exposed to higher afterload. RV can to a certain extent overcome this increased afterload due to IRI by increasing its contractility. The peak contractility tended to be higher in the ventricles that did develop RVF than in those that did not. Second, ventriculo-arterial coupling does not predict RVF before the occurrence of an acute event. Third, the interaction between RV function and lung IRI demonstrated that increased pressure and vascular flow toward the post-ischemic lung aggravated IRI. Ultimately, there was an inverse association between plasma concentrations of troponin-T and the severity of pulmonary IRI. Nonetheless, the occurrence of RVF was not solely determined by troponin-T levels, as both animals with the highest and lowest values experienced RVF.

We have developed a model with a reproducible amount of pulmonary IRI that is reflected in all levels of the assessment (*W*/*D* ratio, physiological, radiological, and morphological assessment) focusing on the factor of mechanical cessation and restoration of blood flow to the lung. Although no actual LTx was performed, the injury is based on warm ischemia and results in the type of reperfusion damage comparable with clinical SSLTx (20, 21). In our model, no recipient immune cells are involved, therefore, the reperfusion injury is triggered mainly by innate immunity. When measuring the cytokine levels in our model, only a difference in IL-6 was observed. This could be influenced by absence of recipient immune cells involvement. However, the short observation period in our experiments might have even stronger impact on this finding. In previous animal work of our group where an actual LTx was performed, we observed that expression of cytokines within the first 6 h after reperfusion was limited (22). Also, in a recent study by Chacon-Alberty et al. (23) focusing on inflammatory markers after LTx the cytokine levels were only measured after 6 h of reperfusion, highlighting the importance of time factor in developing a full inflammatory response. Therefore, we can assume that performing an actual transplantation in our model might have only limited influence on our findings. The aim of our translation model was to focus on the intraoperative events and thus a longer observation period was not considered in this study. The selection of a 1-h time window for subjecting the RV CO to a lung suffering from IRI was determined based on clinical observations regarding the typical duration of performing an anastomosis (24).

We hypothesized that IRI of the pulmonary graft might have a serious impact on RV function, especially when combined with mechanical clamping of the contralateral lung. In the absence of IRI during the initial phase of clamping (*phase 1*), RV was able to resist the increased afterload and did not fail. Only when this mechanical challenge was combined with pulmonary IRI during the last experimental hour (*phase 3*), RVF did occur in 5 of 9 animals. This was accompanied by changes in RV contractility as seen in other large animal models using different means of acute RV afterload increase such as PA banding, balloon inflation in PA, hypoxia, or pharmacologically induced pulmonary hypertension or pulmonary embolism (13, 25–28).

Physiologically, an in-depth analysis of the last experimental hour showed several differences in failing and nonfailing ventricles. First, in the RVF group, a progressive increase in Ea and decrease in SV was observed. Second, bigger Ea increase in the RVF group also led to more pronounced contractility (Ees) increase than in NO-RVF group. We can speculate that the more the homeometric autoregulation is activated, the higher the risk of RVF is. This probably means that ventricles could initially compensate, as was reflected in preserved ventriculo-arterial coupling, until the point of no return. Compared with other researchers studying ventriculo-arterial coupling, our baseline values were lower (<1.0) than expected (26, 28, 29). This might be based on the circumstances of measurement (open chest, open pericardium, and lateral decubitus position). However, a recent study by Kirk et al. (30) showed that open-chest and open pericardium setting in a porcine model does not significantly impair RV systolic function.

Of course, the changes in Ea and Ees could also be present in the NO-RVF group, and we cannot exclude that more ventricles would fail when observing beyond 1 h. Therefore it is plausible that the separation between RVF and non-RVF is rather a matter of time. However, RVF seems to be more related to the severity of lung injury than to time, as the ventricles that failed were exposed to higher Ea.

Our observation that more than 50% of the ventricles fail within 1 h interval is relevant for the implantation time in clinical SSLTx. It is already known that prolonged implantation time is a risk factor for severe PGD development (24). Our results suggest that prolonged implantation might also contribute to the risk of RVF, especially when the transplanted lung suffers from severe IRI. Another clinically relevant observation relates to the practice of test-clamping before lung explanation. Our findings indicate that, based on the progressive decline of stroke volume, we should wait at least 10 min before a decision can be made to proceed with or without ECLS to avoid acute RVF. These findings underline the complexity around the time factor in SSLTx.

Our second hypothesis states that hemodynamic changes occurring during different phases of LTx will have repercussion on the transplanted graft itself. Physiologically, two interesting patterns have been identified in our experimental setting. Both mean PAP and LPA flow during the last experimental hour (*phase 3*) were correlated to IRI severity. This finding is in accordance with Pérez-Terán et al. (31, 32) that showed that better RV function results in higher risk of severe PGD after SSLTx. We can speculate a vicious circle where RV function contributes to IRI, but at the same time the underlying IRI severity might also compromise the RV. Interestingly, the higher flows and pressure for the specific animals could already be observed at earlier time points during the experiment. This could help the clinician to anticipate specific measures reducing flow and pressure toward the newly transplanted graft. Moreover, the existence of this vicious circle is substantiated by a negative correlation observed between troponin-T levels and both the flow directed toward the injured lung and the severity of pulmonary IRI. Specifically, the less the extent of RV ischemic injury, the higher RV CO was generated, subsequently leading to an increase of pulmonary IRI severity.

It remains very difficult to predict RV failure in our model, even when advanced ventricular behavior is assessed by PV-loop analysis. During our experiments, not all ventricles that failed were able to produce high flows and pressures before the failure. The pathophysiological mechanism of RVF due to increased afterload involves ischemia (8, 33). Interestingly, the experiments resulting in RVF were the ones either with the highest or the lowest troponin-T levels of all performed experiments of CLA group. Our findings suggest that the experiments in which RVF occurred, despite the presence of the lowest troponin levels, exhibited the most pronounced manifestation of homeometric autoregulation. This underlines the complexity of RVF mechanisms and unpredictability of this phenomenon.

Specific interventions on flow, pressure, and severity of pulmonary and/or RV injury would be needed to further elucidate the primum agens in this delicate RV-IRI relationship. Furthermore, RV dysfunction and failure is defined as a clinical syndrome without any specific functional cut-off values (8). There are no guidelines on how to apply this definition to the specific acute setting of SSLTx. From a clinical perspective, hemodynamic collapse is often the most obvious sign of RVF, and therefore we have incorporated this translational aspect in our experimental design. Based on the analysis of the available parameters related to LV function and systemic circulation, it was determined that the state of the LV remained stable throughout the experiments. Consequently, it is unlikely that the LV itself was the primary cause of the observed hemodynamic collapse. However, considering the ventricular interdependence, we can anticipate a certain degree of LV involvement in this collapse.

Our findings can contribute to the physiological rationale behind ECLS use during SSLTx. Although the use of ECLS during SSLTx has widely been reported, no clear consensus concerning the indications and the frequency of use have been defined. We know that ECLS devices, especially veno-arterial extracorporeal membrane oxygenation (VA-ECMO), partially divert pulmonary flow away from the pulmonary graft and RV. Our data add a physiological basis that ECLS can protect the lung from further IRI development by reducing the hemodynamic stress. Also, reducing afterload should be beneficial for the RV as a pump.

However, the association between ECLS and PGD incidence and severity has been investigated. While Hoetzenecker et al. (34) relate their low PGD incidence to VA-ECMO use, Loor et al. (35) recently published results from a multicenter international registry showing an association between PGD and VA-ECMO. Also, the invasive nature of ECLS might add additional complications that have to be taken in balance with the hemodynamic and respiratory support. These can occur due to the invasive nature of ECLS, such as cannulation-related vessel injury or wound complications in the cannulation site (36, 37). Furthermore, components of ECLS devices also contribute to the adverse effects, such as hemolysis, bleeding, coagulation disorders, or inflammatory response (23, 38).

As the LTx community calls for further research in this direction, our animal model provides an ideal platform to facilitate it and investigate the effect of innovative ECLS strategies in future studies.

### Limitations

This study has several limitations. First, the interspecies differences between humans and pigs do not allow for full translation of the experimental results. In particular, RL of the pig has an accessory lobe and different upper lobe anatomy. This is why we performed our experiments focusing on the LL, whereas clinically, RL is often transplanted first. Also, in our experiments, only male animals were used due to logistical reasons, and this should be avoided in future studies to strengthen the translation aspect of the results. Second, this model is a surrogate for SSLTx. Although it allows for separate study of the hemodynamic factor in LTx, it also means that the interaction of this factor with all other factors leading to PGD are not addressed. Furthermore, as we were studying the physiology of the RV and IRI interaction we did not try to stop or prevent RV failure by any other means than fluid management and limited dosage of vasopression. This does not correspond with the clinical management that offers more options. In addition, it is conceivable that certain aspects of pulmonary IRI-RV interaction might have been compromised or diminished due to the experimental setup involving the lateral decubitus position, open chest, and open pericardium.

Furthermore, the lungs and RV of the pigs were healthy at baseline, which does not fully represent the clinical situation. Patients undergoing SSLTx often have some degree of pulmonary hypertension caused by the diseased lungs at the beginning of the procedure. Also, given that the primary emphasis of this study revolved around the characterization of hemodynamic alterations, a systematic collection of RV samples for histological evaluation was not performed. Finally, the experimental design is limited by its duration. It focuses on the acute intraoperative events and does not address development of the pulmonary IRI-RV interaction after chest closure.

### Conclusions

We are the first to approach the interaction between RV and IRI in a physiological way with a translational large animal model. Our findings demonstrate a delicate balance between the development of IRI and RV function. In clinical transplantation, both the pulmonary graft and RV might benefit from supportive measures. Our data mainly point toward the physiological benefit of ECLS technology support. At the same time, our model offers a unique platform to investigate innovative ECLS approaches in future studies.

## DATA AVAILABILITY

The data underlying this article will be shared on reasonable request to the corresponding author.

## SUPPLEMENTAL DATA

10.6084/m9.figshare.23498511 Supplemental Fig. S1: https://doi.org/10.6084/m9.figshare.23498511.

10.6084/m9.figshare.23498514 Supplemental Fig. S2: https://doi.org/10.6084/m9.figshare.23498514.

10.6084/m9.figshare.23498541 Supplemental Table S1: https://doi.org/10.6084/m9.figshare.23498541.

10.6084/m9.figshare.23498532 Supplemental Table S2: https://doi.org/10.6084/m9.figshare.23498532.

10.6084/m9.figshare.23498523 Supplemental Table S3: https://doi.org/10.6084/m9.figshare.23498523.

10.6084/m9.figshare.23498526 Supplemental Table S4: https://doi.org/10.6084/m9.figshare.23498526.

10.6084/m9.figshare.23498535 Supplemental Table S5: https://doi.org/10.6084/m9.figshare.23498535.

10.6084/m9.figshare.23498538 Supplemental Table S6: https://doi.org/10.6084/m9.figshare.23498538.

10.6084/m9.figshare.23498529 Supplemental Table S7: https://doi.org/10.6084/m9.figshare.23498529.

10.6084/m9.figshare.23498544 Supplemental Table S8: https://doi.org/10.6084/m9.figshare.23498544.

## GRANTS

This research has been supported by KU Leuven funding: C2 project (C24/18/073).

## DISCLOSURES

No conflicts of interest, financial or otherwise, are declared by the authors.

## AUTHOR CONTRIBUTIONS

M.O., T.V., D.E.V.R., R.V., L.J.C., P.C., and A.P.N. conceived and designed research; M.O. performed experiments; M.O. and A.V. analyzed data; M.O., T.V., P.C., and A.P.N. interpreted results of experiments; M.O. prepared figures; M.O., and A.P.N. drafted manuscript; M.O., T.V., A.E.F., A.V., D.V.B., S.O., J.V.S., J.K., X.J., W.C., S.E.V., G.M.V., B.M.V., D.E.V., R.V., L.J.C., P.C., and A.P.N. edited and revised manuscript; M.O., T.V., A.E.F., A.V., D.V.B., S.O., J.V.S., J.K., X.J., W.C., S.E.V., G.M.V., B.M.V., D.E.V., R.V., L.J.C., P.C., and A.P.N. approved final version of manuscript.
